# Resuscitation Patterns and Massive Transfusion for the Critical Bleeding Dog—A Multicentric Retrospective Study of 69 Cases (2007–2013)

**DOI:** 10.3389/fvets.2021.788226

**Published:** 2022-01-05

**Authors:** Claire Tucker, Anna Winner, Ryan Reeves, Edward S. Cooper, Kelly Hall, Julie Schildt, David Brown, Julien Guillaumin

**Affiliations:** ^1^Department of Clinical Sciences, Colorado State University, Fort Collins, CO, United States; ^2^TetraMed, Fort Collins, CO, United States; ^3^Department of Clinical Sciences, The Ohio State University, Columbus, OH, United States; ^4^Department of Clinical Sciences, The University of Tennessee, Knoxville, Knoxville, TN, United States; ^5^Department of Statistics, Colorado State University, Fort Collins, CO, United States

**Keywords:** exsanguination, massive bleeding, hemorrhagic shock, massive transfusion, ATLS

## Abstract

**Objective:** To describe resuscitation patterns of critically bleeding dogs, including those receiving massive transfusion (MT).

**Design:** Retrospective study from three universities (2007–2013).

**Animals:** Critically bleeding dogs, defined as dogs who received ≥ 25 ml/kg of blood products for treatment of hemorrhagic shock caused by blood loss.

**Measurements and Main Results:** Sixty-nine dogs were included. Sources of critical bleeding were trauma (26.1%), intra/perioperative surgical period (26.1%), miscellaneous (24.6%), and spontaneous hemoabdomen (23.1%). Median (range) age was 7 years (0.5–18). Median body weight was 20 kg (2.6–57). Median pre-transfusion hematocrit, total protein, systolic blood pressure, and lactate were 25% (10–63), 4.1 g/dl (2–7.1), 80 mm Hg (20–181), and 6.4 mmol/L (1.1–18.2), respectively. Median blood product volume administered was 44 ml/kg (25–137.4). Median plasma to red blood cell ratio was 0.8 (0–4), and median non-blood product resuscitation fluid to blood product ratio was 0.5 (0–3.6). MT was given to 47.8% of dogs. Survival rate was 40.6%. The estimated odds of survival were higher by a factor of 1.8 (95% CI: 1.174, 3.094) for a dog with 1 g/dl higher total protein above reference interval and were lower by a factor of 0.6 (95% CI: 0.340, 0.915) per 100% prolongation of partial thromboplastin time above the reference interval. No predictors of MT were identified.

**Conclusions:** Critical bleeding in dogs was associated with a wide range of resuscitation patterns and carries a guarded to poor prognosis.

## Introduction

Critical bleeding, also termed massive hemorrhage or exsanguination, is a major cause of mortality and morbidity in humans and animals (1, 2). Although there is no universal definition of critical bleeding in people, it has been defined as (1) blood loss exceeding circulating blood volume within a 24-h period or (2) blood loss of 50% of circulating blood volume within a 3-h period (3). There is no established veterinary definition for critical bleeding. In people, the principles of treatment for critical bleeding involve early intervention, including hemostatic resuscitation with blood products, damage control surgery, and administration of anti-fibrinolytic agents, among other interventions (1, 4). However, there is a paucity of literature in veterinary medicine regarding critical bleeding. Although not specifically focused on critical bleeding, only one retrospective study has been published, describing massive transfusion (MT) in 15 dogs (5). In that study, acute blood loss due to blunt or penetrating trauma was the most common cause for MT, but hemolysis, coagulopathy, and bone marrow failure were also included, and resuscitation patterns of critical bleeding dogs were not described (5).

MT is most commonly defined as transfusion of blood products of more than a blood volume over 24 h, adapted from the human definition of >10 units of packed red blood cells (pRBCs) in 24 h (2, 5, 6). Recently, more dynamic, time-dependent definitions have been introduced in people (e.g., ≥50% estimated blood volume in 3 h) challenging the traditional, volume-dependent definitions (6, 7). Dynamic definitions focus more on the acute hemorrhage control period, during which decisions have the most clinical impact (7, 8). These dynamic definitions eliminate the concern for survivor bias, where people suffering from critically bleeding may never reach the classic definition due to death before the 24-h cutoff (7). In human medicine, these definitions have been shown to select a cohort of critically bleeding patients with more uniform characteristics, which, if adapted into the veterinary field, may lead to faster recognition and implementation of MT protocols (MTPs) (7).

The primary treatment for exsanguination in both human and veterinary medicine is the transfusion of fresh whole blood (FWB) or a combination of blood components, such as pRBCs and/or fresh frozen plasma (FFP) (5, 6). Recent literature reviews in people on MTPs suggest that the administration of plasma to pRBCs should be in a ratio of 1:1 or 1:2, something that that has not yet been explored in veterinary medicine (1, 9). Veterinary studies investigating data related to when to trigger MTP, how to implement resuscitation of dogs with critical bleeding (including the type of blood products, amount of product give, pRBC/FFP ratio, and ratio of non-blood product resuscitation fluids to blood products), and dosing and timing of additional therapies such as fibrinolytic inhibitors have not yet been performed.

Large-volume transfusion, including MT, has been associated with several complications, including acute or delayed transfusion reactions, hemolysis, hypothermia, hypocalcemia, hypomagnesemia, acidosis, and organ dysfunction (5, 6, 10, 11). When managing the critically bleeding patient, additional considerations for veterinarians may include resource utilization, costs, and critical care of the surviving patients (5). These aspects are an essential part of the conversation between the critical care team and the pet owner.

This study describes the presenting characteristics of critically bleeding dogs, diagnostics, resuscitation patterns, adjunct therapies, and outcome. The primary goal of this retrospective study was to describe resuscitation patterns of critically bleeding dogs, defined as needing 25 ml/kg or more of blood products, to identify prognostic factors. The secondary objective of this study was to investigate the subset of critically bleeding dogs receiving MT. We hypothesized that critical bleeding dogs requiring a MT will have a higher mortality rate compared with critical bleeding dogs not requiring a MT.

## Materials and Methods

Electronic medical records at Colorado State University (CSU), University of Minnesota (UMN), and The Ohio State University (OSU) were searched for dogs who were charged for more than three blood products during a single visit between January 1, 2007, and December 31, 2013. Blood products were defined as pRBCs, FFP, cryoprecipitate (CRYO), cryo-poor plasma (CPP), FWB, or human serum albumin (HSA).

### Inclusion Criteria

Inclusion criteria were critically bleeding dogs, defined as undergoing external or internal blood loss such that medical or surgical intervention was required for survival, that were receiving rapid volume expansion using blood products, defined for this study as needing 25 ml/kg or more of blood products, due to hypovolemic hemorrhagic shock, defined as shock class III or IV using a modified advanced trauma life support (mATLS) classification (Table 1), that was caused by blood loss including, but not limited to trauma, spontaneous hemoabdomen, or surgical procedure (Table 1) (12–17). Decision for mATLS class was made using at least three criteria in the same category, and conflict between categories was resolved using serum lactate level (Table 1). Cases were excluded if blood products were not used for rapid volume expansion, if the dog was in hemorrhagic shock class I or II, or if the medical record was incomplete, especially regarding accurate timing of resuscitation.

**Table 1:** Hemorrhage classification using a grading system modified from the ATLS guidelines (12).

	**Mental State**	**HR**	**Blood pressure**	**Mm color**	**CRT**	**Lactate**
Class I	Normal to slightly obtunded	<120	Normal	Pink	<2 s	<3
Class II	Slight obtunded/depressed	Mild increase > 120	Normal	Pink - Pale	<3 s	3–5
Class III	Moderately obtunded/depressed/weak but ambulatory	Moderate increase > 140	Decreased (SBP < 120 or MBP < 100)	Pale - White	> 3 s	5–10
Class IV	Stuporous/comatose/recumbent	Marked increase > 170	Decreased to not detectable (SBP < 100 or MBP < 65)	White	> 3 s	> 10

*Decision for mATLS class was made using at least three criteria in the same category, and conflict between categories was resolved using serum lactate level*.

### Definitions

MT was defined using classic and dynamic definitions. MT24 was defined as transfusion of blood products in excess of the entire blood volume of the dog, defined in this study as 80 ml/kg of body weight over 24 h (18–20). MT3, MT6, and MT12 were defined as transfusion of blood products in excess of the one-half of blood volume of the dog over 3, 6, or 12 h, respectively (7, 21). Dogs were categorized by the earliest MT classification met. If the critical bleeding happened during surgery, then the dog was considered surgically managed. If the critical bleeding happened after surgery and no additional surgery was performed, then the dog was deemed medically managed. The cause of critical bleeding was divided into four groups: trauma, spontaneous hemoabdomen, intra/perioperative surgical period, and miscellaneous.

### Data Collected

Admission variables collected from each dog included age, body weight, breed, rectal temperature, complete blood count, biochemistry profile, hemostatic testing, blood pressure (BP), level of consciousness, pulse quality, mucous membrane color, and capillary refill time (CRT). The prothrombin (PT) and activated partial thromboplastin time (aPTT) values were calculated as the percent increase over either the high end of normal or the control for each individual device (if the high end of normal was not available). If PT or PTT was above measurable range, then the recorded value was 999. BP measured by Doppler was recorded as systolic BP (22, 23). If BP was reported as undetectable, then the recorded value was 20 mm Hg. The presence of an American College of Veterinary Emergency and Critical Care (ACVECC) diplomate or resident during resuscitation was established if the name was listed as primary clinician on the electronic medical record, the resuscitation admission sheet, or the hospitalization flow sheet.

### Interventions

All blood products used were supplied by a local (i.e., institution-specific) community-based blood bank, collected, and processed using standard blood banking protocols (24–26). Variables recorded for blood products administered included type of products (such as RBC, FFP, FWB, CRYO, CPP, and HSA), volume of products, and time over which products were transfused. Additional blood products given after the period of rapid volume expansion were recorded. Any additional therapy the dog received during the transfusion, including antifibrinolytics, vasopressors/positive inotropes, vitamin K, magnesium infusion, calcium infusion, arginine vasopressin (DDAVP) use, and surgery to control critical bleed, were also recorded. For non-blood products administered during rapid volume expansion, only boluses of isotonic crystalloids, hypertonic saline, and synthetic colloids were recorded. Constant rate infusions during resuscitation efforts were not recorded. Pertinent clinicopathology values after resuscitation, such as HCT, TP, and ionized calcium (iCa), were recorded, if available, although timing related to resuscitation was not. Outcome was defined as survived to discharge, died, or euthanized. Died and euthanized were combined as a single outcome for analysis.

If the exact volume of blood product used was not indicated, then each blood product unit was assigned a standardized volume, which was dependent on individual institutions (Table 2). None of the three institutions carried platelet products at the time of the study. For calculation of volume ratio of RBC to plasma products (i.e., FFP, CRYO, CPP, and HSA), autotransfusion and FWB transfusion were counted as one-half RBC and one-half plasma.

**Table 2:** Standardized blood product volume (per unit of blood product) and blood product availability as organized per institution.

**Blood product volume (ml)**	**CSU**	**OSU**	**UMN**
RBC	350	200	250
FFP	150	100	100
CRYO	NA	70	NA
CPP	NA	200	NA
FWB	450	450	450

*CSU, Colorado State University; OSU, The Ohio State University; UMN, University of Minnesota; RBC, red blood cells; FFP, fresh frozen plasma; CRYO, cryoprecipitate; CPP, cryo-poor plasma; FWB, fresh whole blood; NA, not available. All volumes are in milliliters*.

### Timing of Resuscitation

The start time of the resuscitation of the critically bleeding dog was considered T0. If resuscitation was initiated <10 min after the recognition of a critically bleeding dog, then the time between recognition and start of resuscitation was 0 min. The timing of resuscitation was from the beginning to the end of rapid volume expansion.

### Statistical Analysis

Data normality was assessed using Shapiro-Wilk or D'Agostino-Pearson. Data are presented using median (range) or mean (± standard deviation) as appropriate. Differences in nominal data between subgroups of dogs (defined by baseline characteristics) were tested using Fisher's exact test for categorical data. Continuous data were compared using Mann-Whitney test. For all analyses, the survival status (i.e., discharged or died/euthanized) and presence/absence of MT were separately considered as response variables.

Simple logistic regression models were fit with each predictor (Tables 3, 4) and each response to examine the association between each predictor and response (survival status or presence/absence of MT) and examine with likelihood ratio tests. Log or square root transforms were considered for some predictors that boxplots showed were severely non-normal in distribution. On the basis of Akaike's information criterion, a transformed variable was only preferred for the model with MT as a response and the speed of resuscitation as a predictor, where the log transform was preferred to the untransformed data. Multiple logistic regression models were fit for each response variable starting with all predictors that were significant at α = 0.1 from the simple logistic regression analyses. Because of the missing data, only variables that were measured for at least 70% (*n* = 49) of the dogs and present at all three locations (CSU, UMN, and OSU) were considered for multiple regression. Backward elimination was used to remove predictors until all remaining predictors had *p* < 0.05. All analyses were conducted using a commercially available software^1^^,^^2^.

**Table 3:** Patient baseline characteristics and clinicopathological data.

**Variable**	**All Dogs (*n =* 69)**	**Survivors (*n =* 28)**	**Non-Survivors (*n =* 41)**	***p*-value**
**Institution** ^**!**^				0.545
Colorado State University *(n)*	26 (37.7)	12 (46.1)	14 (53.9)	
The Ohio State University *(n)*	29 (42.0)	12 (41.4)	17 (58.6)	
University of Minnesota *(n)*	14 (20.2)	4 (28.6)	10 (71.4)	
**Reasons for Bleeding** ^**!**^				0.901
Traumatic *(n)*	18	6	12	
Bleeding during/after surgery *(n)*	18	8	10	
Miscellaneous *(n)*	17	7	10	
Spontaneous hemoabdomen *(n)*	16	7	9	
**Signalment** ^**!**^	
Age (years)	7 (0.5–18)	6.5 (0.5–14)	7 (0.8–18)	0.464
Weight (kg)	20.0 (2.6–57.0)	20.0 (3.0–57.0)	20.0 (2.6–52.0)	0.775
mATLS Score III *(n)*	31	12	19	0.775
mATLS Score IV *(n)*	38	16	22	
**Admission parameters**	
Temperature (°F)	99.0 ± 2.2	98.2 ± 2.1	98.8 ± 2.1	
Heart Rate (bpm)	172 (40–250)	166 (104–240)	179 (40–250)	
Respiratory Rate (bpm)	36 (6–140)	32 (20–80)	38 (6–140)	
**Blood pressure before pressors** ^**!**^	
Systolic blood pressure (mmHg)	80 (20–181)	61 (20–115)	94 (20–181)	0.007
Mean blood pressure (mmHg)	77 ± 21	68 ± 22	82 ± 20	0.087
**Complete blood count**	
Hematocrit^!^ (%)	25 (10–63)	22 (10–58)	28 (12–63)	0.540
Platelet count^!^ (×10^∧^9/L)	96 (18–546)	101 (25–546)	94 (18–539)	0.587
Proportion of thrombocytopenic dogs (%)	62.5	45.5	71.4	
**Serum biochemistry** ^**!**^	
Total protein (g/dl)	4.1 (2.0–7.1)	5.0 (2.0–7.1)	3.8 (2.0–7.0)	0.033
Ionized calcium (mg/dl)	4.75 (2.33–5.37)	4.79 (4.27–5.29)	4.72 (2.33–5.37)	0.686
Ionized magnesium (mg/dl)	1.37 ± 0.31	1.42 ± 0.19	1.34 ± 0.37	0.524
Lactate (mmol/L)	6.4 (1.1–18.2)	5.8 (1.4–18.2)	7.3 (1.1–15.1)	0.347
**Coagulation** ^**!**^	
PT (% of high end of normal or control)	155 (64–1,558)	156 (62–1,377)	152 (82–1,558)	0.733
aPTT (% of high end of normal or control)	176 (37–999)	118 (67–343)	245 (78–999)	0.008

*Normally distributed data are presented as means ± standard deviation. Non-normally distributed data are presented as median (range). P-values are from simple logistic regression. PT, prothrombin time; aPTT, activated partial thromboplastin time; mATLS, modified advanced trauma life support*.

! *Variable included in the logistic regression analyses*.

**Table 4:** Baseline characteristics, outcome and intervention on critically bleeding dogs receiving or not a massive transfusion, with *p*-values based on simple logistic regression analysis.

**Variable**	**All dogs (*n =* 69)**	**Massive transfusion (*n =* 33)**	**No massive transfusion (*n =* 36)**	***p*-value**
**Institution** ^**!**^				0.023
The Ohio State University *(n)*	29	13	16	
Colorado State University *(n)*	26	17	9	
University of Minnesota *(n)*	14	3	11	
**Reasons for bleeding** ^**!**^				0.309
Traumatic *(n)*	18	7	11	
Bleeding during/after surgery *(n)*	18	12	6	
Miscellaneous *(n)*	17	7^*^	10^#^	
Spontaneous hemoabdomen *(n)*	16	7	9	
**Blood products** ^**!**^	
Total blood products (ml/kg)	44 (25–137.4)	56 (40–137.4)	34 (25–70)	NA
Ratio plasma: RBC (ml)	0.8 (0–4)	0.8 (0.19–4)	0.7 (0–2)	0.223
Ratio crystalloid: blood product (ml)	0.5 (0–3.6)	0.3 (0–1.8)	0.8 (0–3.6)	0.008
Speed of resuscitation (ml/kg/h)	7.9 (1.5–54.0)	13.4 (4.0–54.0)	3.8 (1.5–30.8)	NA
**Outcome**	
Survival to discharge *(n)*	28	12	16	
**Other interventions**	
Surgical intervention^a!^ (%)	65.2	69.7	61.1	0.454
Vasopressors^b^ (%)	50.0	46.7	54.2	
Aminocaproic acid^b!^ (%)	27.8	26.7	29.2	0.839
Vitamin K^b^ (%)	20.4	16.7	25.0	
Ca2+ supplementation^b^ (%)	7.4	13.3	0	
Mg2+ supplementation^b^ (%)	5.5	3.3	8.3	
DDAVP^b^^,^^d^ (%)	5.5	6.7	4.2	
**Post-transfusion clinicopathologic abnormalities** ^**c**^	
Hematocrit below reference range (%) (*n =* 35)	94.3	88.9	100	
TP below reference range (%) (*n =* 33)	93.9	93.7	94.1	
Lactate above reference range (%) (*n =* 21)	76.2	75	80	
Prolongation of clotting times (%) (*n =* 14)	78.6	85.7	71.4	
Platelet count below reference range (%) (*n =* 7)	100	100	100	
Magnesium below reference range (%) (*n =* 13)	46.1	57.1	33.3	
Ionized calcium below reference range (%) (*n =* 14)	35.7	57.1	14.3	
**Timing of resuscitation** ^**c**^	
Duration of resuscitation (h) (*n =* 67)	7 (1–36)	6 (1.5–24)	8.25 (1–36)	
Time to surgery^!^ (h) (*n =* 37)	3 (0–84)	3.5 (0–84)	3 (0–20)	0.184
Time from admission to transfusion^!^ (h) (*n =* 53)	2 (0–32)	2.5 (0–32)	2 (0–24)	0.134
**MT definition**	
3 h (MT3)	15^$^	15^$^	-	
6 h (MT6)	5	5	-	
12 h (MT12)	12^$^	12^$^	-	
24 h (MT24)	1	1	-	

a *Data available in 69 dogs*.

b *Data available in 54 dogs*.

c *Number in parenthesis represents the number of dogs with available data*.

d *Causes of critical bleeding for dogs receiving DDAVP (n = 3): intra/perioperative surgical period, gastric-dilation and volvulus, and trauma*.

! *Included in logistic regression analyses. Analyses also considered whether the ACVECC diplomate or resident was present*.

* *Miscellaneous causes for MT: anticoagulant rodenticide (n = 4), liver failure (n = 1), mesenteric torsion (n = 1), and aortic laceration post-cystocentesis (n = 1)*.

# *Miscellaneous causes for no MT: anticoagulant rodenticide (n = 4), gastric dilatation and volvulus (n = 3), unknown (n = 2), and pyometra (n = 1)*.

$ *Two dogs in the MT3 and four in the MT12 also fit the MT24 definition but were counted in the earliest category*.

*RBC, red blood cell; TP, total proteins; DDAVP, 1-desamino-8-D-arginine vasopressin; MT, massive transfusion*.

## Results

### Population Baseline Characteristics

Sixty-nine dogs met the inclusion criteria. Twenty-nine cases (42.0%) were from OSU, 26 (37.7%) from CSU, and 14 (20.3%) from UMN (Table 3). Median (range) age was 7 years (0.5–18), and median body weight was 20.0 kg (2.6–57). Age was not associated with survival (*p* = 0.464) or need for MT (*p* = 0.842). Body weight was not associated with survival (*p* = 0.775) or need for MT (*p* = 0.092). Breeds with more than two individuals included 12 mixed breed dogs, nine Labrador Retrievers, six Golden Retrievers, four Dachshunds, three Border Collies, three Miniature Doberman Pinschers, three Shih Tzus, and two of the following breeds: Alaskan Husky, Australian Shepherds, Boston Terriers, Chihuahuas, Miniature Poodles, Standard Poodles, and Yorkshire Terriers. The proportion of dogs experiencing mATLS class III and class IV hemorrhage was 45 and 55%, respectively. Trauma and intra/perioperative surgical period were the most common sources of critical bleeding, with 18 (26.1%) of dogs in each category. This was followed by miscellaneous causes (17 dogs, 24.6%) and spontaneous hemoabdomen usually secondary to bleeding mass (16 dogs, 23.1%) (Table 4). Miscellaneous causes included anticoagulant rodenticide toxicity (*n* = 8), gastric dilatation and volvulus (*n* = 3), unknown (*n* = 2), pyometra (*n* = 1), liver failure (*n* = 1), mesenteric torsion (*n* = 1), and aortic laceration post-cystocentesis (*n* = 1).

Admission variables including temperature, heart rate, and BP before intervention and clinicopathological data are displayed in Table 3.

### Interventions

Overall, the dogs in this study received a median of 44 ml/kg (25–137) of blood products (Table 4). There were no differences in transfused blood volumes between large dogs (i.e., ≥20 kg) and smaller dogs (i.e., <20 kg): 43.0 (25.0–109.0) and 44.0 (25.0–137.0), respectively (*p* = 0.610). The breakdown of blood product per institution and survival status is presented (Table 5). The median time of transfusion was 7.0 h (1–36). Thirty-three dogs met the definition for MT, six at more than one time point. The breakdown of cases fitting each of the various MT definitions was 15, 5, 12, and 1 for MT3, MT6, MT12, and MT24, respectively (Table 4). Two dogs in the MT3 and four in the MT12 also fit the MT24 definition but were counted in the earliest category. Median time from admission to transfusion was 2.0 h (0–32, *n* = 53). A variety of transfusion protocols were used to resuscitate critically bleeding dogs. Median ratio of plasma volume (milliliters) to RBC (milliliters) was 0.8 (0–4.0). The median ratio of non-blood products, which included crystalloids and artificial colloids, to blood products was 0.5 (0–3.6, *n* = 53). Median volume of blood product divided by duration of MT was 7.9 ml/kg/h (1.5–54.0, *n* = 67) (Table 4).

**Table 5:** Number of dogs receiving various blood products, by institution and survival status.

	**OSU**		**CSU**		**UMN**	
	**Survivor**	**Non-survivor**	**Survivor**	**Non-survivor**	**Survivor**	**Non-survivor**
Outcome *(n)*	12	17	12	14	4	10
Received RBC *(n)*	12	17	7	7	3	10
Received FFP *(n)*	12	17	11	11	3	10
Received CRYO *(n)*	3	2	NA	NA	NA	NA
Received CPP *(n)*	1	1	NA	NA	NA	NA
Received HSA *(n)*	0	1	0	1	0	0
Received FWB *(n)*	0	1	8	11	1	1
Received Autotransfusion *(n)*	0	3	0	1	1	2

*Data are presented in number of dogs receiving an individual blood product (e.g., 14 dogs from CSU received RBC, seven in the survivor group, and seven in the non-survivor group). OSU, The Ohio State University; CSU, Colorado State University; UMN, University of Minnesota; RBC, red blood cell; FFP, fresh frozen plasma; CRYO, cryoprecipitate; CPP, cryo-poor plasma; HSA, human serum albumin; FWB, fresh whole blood*.

Forty-five (65.2%) dogs required surgical intervention to control critical bleeding. Median time from massive bleeding to surgery was 3.0 h (0–84). Several other interventions were pursued for a subset of the dogs, and those variables were collected in 54 dogs. Other interventions (% of cases) included vasopressors (50%), aminocaproic acid (27.8%), vitamin K supplementation (20.4%), calcium supplementation (7.4%), magnesium supplementation (5.5%), and DDAVP (5.5%) (Table 4).

### Outcomes

Twenty-eight dogs survived to discharge, making the survival rate 40.6%. Of all the non-survivors, 25 were euthanized and 16 died. Reasons for euthanasia were not recorded in this study. Survival distribution was 12 dogs in the MT group and 16 in the no-MT group, making the survival rates 36.4 and 44.4%, respectively.

Clinicopathologic abnormalities that remained or occurred after transfusion were recorded in a portion of the studied dogs. Of those, 94.3% had a hematocrit below reference range, 93.9% had a TP below reference range, 76.2% had lactate above reference range, 78.6% had prolongation of clotting times, 100% had a platelet count below reference range, 46.2% had magnesium below reference range, and 35.7% had ionized calcium below reference range (Table 4). Whether the case was supervised by an American College of Veterinary Emergency and Critical Care (ACVECC) resident or diplomate was recorded in the medical record for 54 dogs. Of these, 27 (50%) were supervised by ACVECC diplomates or residents.

In the simple logistic regression analysis for survival, blood product volume (*p* = 0.082), total protein (TP) (*p* = 0.033), aPTT (*p* = 0.008), systolic BP (*p* = 0.007), and mean BP (*p* = 0.087) showed a *p* ≤ 0.1. Surgical intervention (*p* = 0.517), use of aminocaproic acid (*p* = 0.683), causes of bleeding (*p* = 0.901), and receiving a MT (*p* = 0.494) did not have a *p* ≤ 0.1 and thus were not found to be predictive of survival. Of the variables with *p* ≤ 0.1, blood product volume, TP, systolic BP, and aPTT met the sample size criteria and were considered in the multiple logistic regression. TP (*p* = 0.007) and aPTT (*p* = 0.011) were found to be predictors of survival after backward elimination. Specifically, the estimated odds of survival were higher by a factor of 1.84 (95% CI: 1.17, 3.09) for a dog with 1 g/dl higher TP above reference interval and were lower by a factor of 0.62 (95% CI: 0.34, 0.92) per 100% prolongation in aPTT above the reference interval. Receiving or not a MT was not predictive of survival (*p* = 0.494). Because of the finding linking odds of survival and aPTT, non-survivor analysis was performed for the subgroup of dogs suffering from anticoagulant rodenticide toxicity (*n* = 8). Their survival was 75% compared with 36% survival for dogs bleeding from other causes (*p* = 0.055).

In the simple logistic regression analysis for MT, blood product volume (*p* < 0.001), speed of resuscitation (*p* < 0.001), crystalloid-to-blood product ratio (*p* = 0.008), as well as presenting characteristics body weight (*p* = 0.092), institution (*p* = 0.023), lactate (*p* = 0.038), iCa (*p* = 0.023), and platelet count (*p* = 0.033) showed a *p* ≤ 0.1 and were included in the final model. Of these, blood product volume, speed of resuscitation, institution, body weight, and lactate met the sample size criteria and were included in the final model. As expected, blood product volume (*p* < 0.001) and speed of resuscitation (*p* < 0.001) were associated with MT. No presenting characteristics that were predictors of MT were found in the multiple logistic regression model after backward elimination.

## Discussion

Our study is the first to describe resuscitation patterns in dogs with critical bleeding. We found a heterogenicity of resuscitation patterns on the basis of wide ranges and standard deviations of interventions used in critically bleeding dogs. Our study identified TP and PTT as prognostic indicators for survival in this patient population, but no difference was found in survival between dogs who received a MT compared with dogs for which blood product resuscitation did not meet our criteria for MT. Furthermore, we were not able to identify predictors for the need to provide MT in our study population.

Our study elected to not only focus on dogs receiving a MT but also chose to include dogs in severe hypovolemic hemorrhagic shock who received a large amount of blood products (i.e., >25 ml/kg) for resuscitation. This was different than the only published study in dogs on MT, which defined MT as transfusion of a volume of blood products in excess of the blood volume of the dog in 24 h or a transfusion of a volume of blood products in excess of one-half of the blood volume of the dog in 3 h (5). Despite this difference, breed, sex, and body weight in our study were similar to the previously published study focusing on MT (5). However, the population in our study had subjectively more dogs characterized as trauma and peri-surgical bleeding when compared to case numbers in the previous study (5).

Trauma and perioperative complications were the most common causes of critical bleeding in our study. This is somewhat consistent with data in people, where surgeries, including cardiothoracic and gastrointestinal surgery, are the most common causes of MT (27, 28). Despite being the focus of most of the research, patients with trauma actually receive <20% of all MT in people (7, 27–29).

To focus on identifying exsanguinating patients, our study used a mATLS score of III or IV as an inclusion criterion. The ATLS score has been commonly used in people since 1978 and has been shown to predict survival need for transfusion in some populations, although recently has come under scrutiny for overestimating the degree of shock (30–33). Although the score is commonly used in people, it is not widely used in veterinary medicine. As ATLS scores I and II are associated lower mortality and rate of transfusion compared with ATLS scores III or IV in people, our study excluded dogs with a mATLS score of I or II (32). Our study did not find a difference in survival between mATLS III and IV, which is consistent with data in people (32).

Although the traditional definition of MT in people is the transfusion of 10 units of red blood cells within 24 h, due to the dynamic nature of critical bleeding, other more dynamic definitions exist, including transfusion of one entire blood volume within 24 h, transfusion of >4 units of pRBCS in 1 h when on-going need is foreseeable, and replacement of 50% of total blood volume within 3 h (2, 34, 35). Because veterinary patients have a wide range of body weights and blood product unit volumes, a definition of veterinary MT utilizing milliliters per kilogram delivered was crafted (5, 17). Our study showed that most of the dogs meeting the MT criteria were not meeting the traditional definition but the dynamic definitions (7). This distinction may help establish veterinary MT definitions in the future. Similarly, six of the dogs studied met several definitions of MT. Although the median blood products volume delivered in our study was 44 ml/kg, about 50% met the MT criteria, and the overall range varied from 25 to 137.4 ml/kg.

In people, the most commonly recommended ratio of plasma products to pRBCs is 1:1 (1). This is close to the ratio found in our study of 0.8, which may reflect the standard of care practiced in large university-based referral centers with boarded clinicians and access to a blood bank. In this ratio, we included the use of autotransfusion and FWB transfusion, as one-half plasma and one-half pRBC, to account for that clinical practice common in veterinary medicine but less common in people (36, 37). By contrast, previously reported data on MT dogs showed an average of pRBC (66.5 ml/kg) and FFP (22.2 ml/kg) delivered, therefore, a 0.3 ratio, although that specific data are not provided (5). Similarly, only 0.5 ml of crystalloid was used for each milliliter of blood product use in our study. This is consistent with the principles of damage control resuscitation that recommends crystalloids restriction, although damage control resuscitation is not a practice that has been widely adopted in veterinary medicine (38).

We report a median total transfusion time of 7.0 h, which is mildly shorter than the mean of 8.5 h previously reported for canine MT (5). Although it is challenging to compare because of the difference in distribution type of this variable, it can also be related to our study focusing on a larger group of critical bleeding dogs, and not only on MT. Similarly, about 65.2% of the dogs in the present study required surgical intervention to control critical bleeding, contrasting to the study on MT who showed that 80% of dogs required surgery (5).

The use of adjunct therapies around the resuscitation efforts was also evaluated. Our study showed a 27.8% use of the antifibrinolytic ACA, compared with that data not being reported in the MT study in dogs (5). Although both ACA and tranexamic acid (TXA) have been used for trauma resuscitation in both dogs and people, ACA was the only antifibrinolytic used in the study. Similar to another retrospective study in dogs, we did not find an association between ACA use and survival (39). This contrasts with people, where TXA is a common addition to MTPs (40). That difference may be explained by our study design including all dogs suffering from critical bleeding or our relatively small sample size compared to studies in people.

Vasopressors, a common treatment for septic shock, were used in 50% of our cases. The addition of vasopressors during resuscitation of critical bleed in human medicine is controversial (41). In a recent randomized control trial, the addition of vasopressin did not result in higher complication rates or mortality, although they were started after definitive hemorrhage control was achieved (42). The utility of vasopressors during critical bleeding resuscitation has not been documented in dogs.

Vitamin K was administered in 11 of 54 (20.4%) of the studied dogs as part of their resuscitation efforts, presumably because of suspected vitamin K antagonism or deficiency. Vitamin K therapy improves hemostasis in these circumstances, and eight of the dogs were known to have suffered from vitamin K antagonism intoxication (43). This is the first time vitamin K supplementation has been reported as a transfusion adjunct in dogs, although it is used in people taking anticoagulant or antiplatelet agents and receiving MT (44).

Our study confirmed previous results of hypocalcemia and hypomagnesemia occurring post-transfusion in about half of dogs (35.7 and 46.1%, respectively), after resuscitation (5). This is due to its chelation by citrate added to blood products to prevent coagulation (45, 46). This is consistent with data in people, where hypomagnesemia and hypocalcemia are well-documented consequences of MT, reported in up to 71 and 97%, respectively, in one study, with the presence of hypomagnesemia often linked to the degree of hypocalcemia (47). Although magnesium is not routinely supplemented, calcium supplementation is a commonly used adjuvant therapy in MT in people, with routine point-of-care monitoring (45, 48).

This is the first time that DDAVP is reported as a pro-hemostatic adjunct for critical bleeding resuscitation in veterinary medicine. The utility of DDAVP or desmopressin has been demonstrated in dogs for treatment of platelet dysfunction such as Von Willebrand disease, and a meta-analysis has shown that its use in people can slightly reduce blood loss and transfusion requirements (49, 50).

Survival rate in our study was 40.6%, with survival rate for MT dogs being 36.4%, which is higher compared to the 26.7% survival from the study of Jutkowitz et al. (5), but similar to the range of 30–69% reported for outcomes of the dogs after transfusion regardless of blood products given (5, 51, 52). Differences in studied populations, especially severity of diseases, assessed as difference in hyperlactatemia, and thrombocytopenia may explain a difference in survival (5). In people, survival depends on the cause of critical bleeding, but survival from MT ranges from 17 to 84%, dependent on the study population, cause of critical bleeding, and units of blood products transfused (53–56).

Our study is the first one documenting clinicopathological abnormalities associated with critical bleeding and transfusion in dogs. Although our study only recorded if the variables were below reference ranges instead of specific numerical values, it showed that many of the dogs on our study suffered from continued anemia, thrombocytopenia, hypomagnesemia, and/or hypocalcemia. Those findings are also commonly documented in people (57).

Our study demonstrated that the estimated odds of survival were higher for a dog with 1 g/dl higher TP above the reference range and lower per 100% prolongation of aPTT above the reference range, which has not been demonstrated before in human or veterinary studies. High total protein may be another indicator of stability in hemorrhagic shock and need for blood products. Prolonged aPTT may show a higher burden of trauma-induced coagulopathy, leading to an increased need for additional blood products and supportive care (58). aPTT is a conventional coagulation assay that measures the clotting activity of the intrinsic pathway cascade, testing the function of all clotting factors except factor VII and factor XIII (fibrin stabilizing factor) (59). aPTT has been documented in many studies as a more sensitive indicator of coagulopathy than PT, although this value should be interpreted with other coagulation factors and viscoelastic testing (60, 61).

Predictors for the need to administer MT among the presented characteristics could not be identified, which may not be surprising considering the groups of critical bleeding dogs who received a MT and the ones who did not were overall similar. It is important to remember that our study focused on dogs with severe hemorrhagic shock who received at least 25 ml/kg of blood products. Therefore, it is possible that criteria such as a dog with a mATLS score of III or IV may require large amount of blood products as part of their resuscitation efforts. Criteria identified in people as predictive for MT include various scoring systems such as the Trauma-Associated Severe Hemorrhage Score, the Assessment of Blood Consumption Score, and the MT Score (62–64). Scoring systems have shown to have a high sensitivity and specificity in predicting if a patient needs a MT when compared to physician clinical decision-making (62, 65).

In our study, we did not use a standardized MTP. Using MTP as part of resuscitation efforts has been a trend over the past 10 years in trauma centers in people (66, 67). Although there is not a universally utilized MTP, most trauma centers have MTPs in place for the 5% of people requiring them (4, 6). Implementation of these protocols for the critically bleeding people, along with improved adherence, showed a 52% mortality reduction in one study (4). These protocols mostly rely on staff training, activation recognition, pre-thawed plasma, amount of packaged blood products, and use of “universal donor” RBC (6). These protocols are not based on goal-directed therapy or titration to a prefixed hemodynamic endpoint but instead focus on rapid recognition and activation of the MTP, blood products management, and adjunct therapy (3).

There are several limitations of this study. Its retrospective nature meant many variables were either not available for all dogs, and treatment protocols were not controlled. This was counterbalanced by the multicenter nature of the study, allowing more dogs to be analyzed. However, there was some heterogenicity between sites, as the proportion of CSU of MT was tripled the one of UMN. This may represent regional caseload, blood product availability, or preference differences of the clinicians. The blood products used also reflected the variety of transfusion products available at each institution, with only OSU carrying CRYO and CPP. It is also possible that decision-making processes were influenced by economic circumstances.

Our study leverages the number of enrolled cases through multicentric design to allow for simple and multiple logistic regression for all causes of critical bleeding. Subgroup analysis would only allow for non-survivor characteristics analysis and is therefore beyond the scope of our study. Although it is customary in veterinary to combine dogs who were euthanized and died in a single non-survivor group, the number of euthanized dogs in our study may represent a substantial bias. However, many dogs in our study received aggressive and expensive treatments, and reasons for the high euthanasia proportion in our study may include humane euthanasia due to futility or impeding cardiac arrest. In addition, the scope and decision-making around surgical stabilization could have varied among the three institutions, potentially affecting surgical outcomes. For all retrospective studies, medical records may have been missed appropriate charging or use of blood products. Our study also focused on critical bleeding dogs and not on MT, and the initial exclusion of dogs only receiving two blood transfusion products may have skewed the dog population toward larger breed dogs, although we had several small dogs in our data set. Last, the mATLS classification is not currently validated in dogs despite decades of research in people.

## Conclusions

This retrospective study characterized a population of critically bleeding dogs, including those receiving MT, and identified TP and aPTT as prognostic indicators for survival. Further prospective studies are needed to establish MTPs and when to trigger a MT based on the admission data. A translational lens to these studies will allow development of a natural animal model that can inform human literature on critical bleeding, exsanguinating injuries, and MT.

## Data Availability Statement

The raw data supporting the conclusions of this article will be made available by the authors, without undue reservation.

## Author Contributions

JG, AW, JS and KH: concept, design, and data collection. JG, EC, CT, AW, RR, and DB: data analysis and manuscript draft. All authors contributed to critical revisions of the manuscript.

## Conflict of Interest

The authors declare that the research was conducted in the absence of any commercial or financial relationships that could be construed as a potential conflict of interest.

## Publisher's Note

All claims expressed in this article are solely those of the authors and do not necessarily represent those of their affiliated organizations, or those of the publisher, the editors and the reviewers. Any product that may be evaluated in this article, or claim that may be made by its manufacturer, is not guaranteed or endorsed by the publisher.

## Footnotes

^1^R Core Team (2019). R: A Language and Environment for Statistical Computing. R Foundation for Statistical Computing, Vienna, Austria. Available online at: https://www.R-project.org/.

^2^MedCalc. Statistical Software Version 20.011. MedCalc Software bvba, Ostend, Belgium.
